# Quantifying the impact of vaccination on pertussis dynamics in Sweden

**DOI:** 10.1371/journal.pcbi.1014538

**Published:** 2026-08-04

**Authors:** Tobias S. Brett, Andrew Tredennick, Michael Briga, Laurent Coudeville, Denis Macina, Matthieu Domenech de Cellès, Pejman Rohani

**Affiliations:** 1 Odum School of Ecology, University of Georgia, Athens, GeorgiaUnited States of America; 2 Center for the Ecology of Infectious Diseases, Odum School of Ecology, University of Georgia, Athens, GeorgiaUnited States of America; 3 Metrum Research Group, Boston, Massachusetts, United States of America; 4 Infectious Disease Epidemiology Group, Max Planck Institute for Infection Biology, Berlin, Germany; 5 Department of Biology, University of Turku, Turku, Finland; 6 Turku Collegium for Science, Medicine and Technology, University of Turku, Turku, Finland; 7 PandemiX Center of Excellence, Roskilde University, Roskilde, Denmark; 8 Charité Centre for Global Health, Charité Universitaetsmedizin, Berlin, Germany; 9 Global Medical, PPH Franchise, Sanofi, Lyon, France; 10 Department of Infectious Diseases, College of Veterinary Medicine, University of Georgia, Athens, GeorgiaUnited States of America; University of Hong Kong, HONG KONG

## Abstract

The last few decades have witnessed a resurgence in pertussis notifications in a number of countries with high vaccine coverage, including Sweden. The underlying causes of the resurgence have been the subject of much scientific debate. To arbitrate among the putative drivers of the resurgence in Sweden, we formulated a mechanistic transmission model which we fit to age-structured time-series notifications data via likelihood maximisation. Given our model, we find the data are best explained by the combined effects of a low basic reproductive number, incomplete (leaky) DTaP-derived immunity, a much lower reporting probability of infections in older individuals and waning of vaccine-derived immunity. In addition, our modelling explains the post-2014 resurgence as a combination of two factors. First, a dynamical transient known as the honeymoon effect, in which a rebound in transmission follows after a rapid decrease in the average population susceptibility. Second, an increase in the infection reporting probability from 2014 onwards, likely due to the use of new laboratory testing methods. Additionally, we used our fitted transmission model to reconstruct indirect protective effects of vaccination. Our results suggest immunization prevents about 50% of potential infections in individuals too young to receive vaccination. However, our statistical inference demonstrates that pertussis elimination is not possible with routine immunization using acellular vaccines due to the combined effects of vaccine leakiness and waning immunity.

## Author summary

The recent resurgence in cases of pertussis, in the face of high vaccine coverage has puzzled scientists. A number of mechanisms have been postulated as explanations, including that: i) protective immunity may only last for a limited duration, ii) vaccination may only provide incomplete protection, allowing for breakthrough infection and iii) that improvements in disease surveillance (including new testing methods) may have increased the proportion of infections that are detected. To understand the role of these mechanisms in driving the resurgence, we undertook a mathematical modelling study using data from Sweden. We fitted our model to time-series data of lab-confirmed pertussis cases using likelihood-based inference. Given our model, our results suggest that all the above mechanisms were implicated in the Swedish pertussis resurgence. Specifically, we found the data were best explained by incomplete and transitory DTaP-derived immunity, a low basic reproductive number, a low detection probability for pertussis infections in older individuals and an increase in the probability of detection from 2014 onwards. Finally, our results also suggest the role of the “honeymoon effect”: a transient dynamical phenomenon whereby a sudden increase in population immunity causes an rapid initial drop in the transmission rate, however transmission later rebounds.

## Introduction

Pertussis is an infectious disease caused by the bacterium *Bordetella pertussis* [1–4]. The pathogen is considered highly infectious and can be fatal, especially in very young infants [5,6]. Vaccines have been demonstrated to effectively protect against severe disease [7–10], prompting a number of countries to adopt immunization programs from the 1950s onwards [7,11–16]. A key challenge in reducing the burden of pertussis morbidity and mortality is that many high-risk infants are too young to receive vaccination, leaving them vulnerable to infection [13]. Strategies to protect these individuals include maternal vaccination [17,18], which provides passive protection and reduces risks of mother-infant transmission [19], and indirect protection achieved by reducing pertussis circulation in the wider population through routine immunization [4,20–23].

While immunization was initially successful at driving down pertussis incidence [7,12,14,20,24,25], in recent decades a number of countries with high coverage have witnessed a pronounced rebound in pertussis notifications, including the US [16,26,27], UK [10,28,29], Mexico [30], Norway [31] among others [4,23,32]. This has stymied efforts at achieving herd immunity, and has generated much debate regarding the putative drivers of resurgence [27,33–36]. Hypothesised, but not mutually exclusive, drivers include:

1. Transient vaccine-derived immunity, *i.e.,* protection wanes over time [37–41]; evidence comes from modelling studies [27,28,42], frequency-matched studies [41], and clinical observations [37,38,43];
2. Incomplete or imperfect protection, also known as vaccine “leakiness,” [44] which occurs when vaccine-induced protection is mismatched with the circulating bacterium [34,45,46], allowing for breakthrough infections while still providing some measure of protection against severe disease but perhaps also allowing for secondary transmission (while simultaneously reducing infection ascertainment rates); evidence comes from studies of temporal trends in the frequency of pertactin-deficient isolates from Europe and the increasing mismatch between allelic variants in *B. pertussis* isolates and vaccine components [47]; and
3. Improvements in disease surveillance, including new molecular diagnostics such as PCR tests [48], causing an increase in disease notifications; some have proposed that this may be the sole explanation for the rise in notifications, and that there is not an underlying increase in pathogen transmission [9].

To explore the compatibility of the above mechanisms with observed pertussis transmission patterns, we examined notifications in Sweden, which provides a data-rich study system [21,49]. After a 17-year hiatus in pediatric immunization due to concerns about whole-cell pertussis vaccine safety [50], the acellular pertussis vaccine was added to the routine schedule in 1997 as part of the diphtheria, tetanus and acellular pertussis (DTaP) vaccine [51]. Given its inclusion within an already well-established vaccination program, immunization uptake has remained above 97% from 1997 onwards [52].

To understand the drivers of the recent resurgence in notifications, and its implications for vaccination effectiveness, we undertook a mathematical modelling study. We formulated a mechanistic, stochastic, age-structured transmission model that accounted for various modes of vaccine failure (listed above). Within the model, specific parameters accounted for the different modes of vaccine failure. Values for these unknown parameters were estimated using likelihood-based inference [27,53]. Our model also accounted for age-stratified patterns of contact [21,54,55] and age-specific reporting. We found that the data are best explained by a small reproductive number, intermediate values for the vaccine protection parameter, and waning immunity. Despite near-universal routine immunization coverage, our findings indicate that elimination is impossible to achieve with the current generation of vaccines due to the volume of breakthrough infections, which stem from a combination of vaccine leakiness and waning immunity. Furthermore, results suggest that these breakthrough infections are much less likely to be reported. That said, while elimination is at present not feasible, we do find evidence for indirect protection of unvaccinated newborns due to reduced pertussis prevalence. These results highlight key challenges in pertussis modelling, in particular in disentangling temporal changes in the surveillance process (and hence the reporting probability) from trends in the underlying transmission process.

## Results

**Fig 1:**
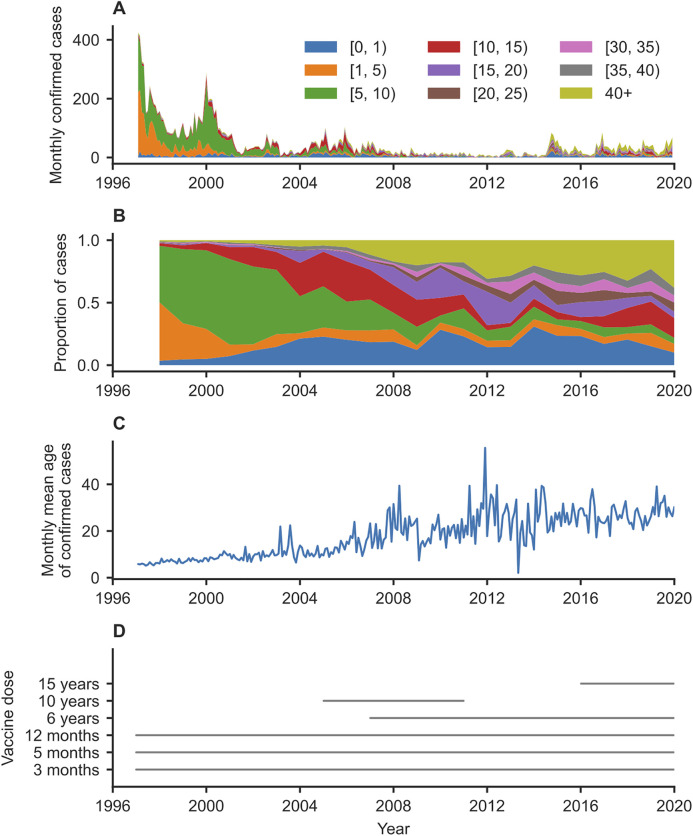
Pertussis cases and vaccine schedule in Sweden. A) Annual laboratory test confirmed cases of pertussis from 1997 until 2020 by age group. After the introduction of routine vaccination in 1997, confirmed cases in individuals eligible for vaccination dropped rapidly (age groups [1,5) years and [5, 10) years). The decline in cases among the at-risk [0, 1) years age group was less pronounced. Post-2014 there was an apparent increase in notifications for both the [0,1) and 10+ age groups. B) Proportion of confirmed cases by age group. Following the introduction of vaccination, confirmed cases showed a pronounced shift to older ages. C) Mean age of confirmed cases by month. D) Vaccine schedule in Sweden. Beginning in 1997, 3-dose primary vaccination with DTaP was added to the vaccine schedule. From 2005 onwards, booster doses were added to the schedule: first at 10 years (DTaP; 2005–2011), then 6 years (DTaP; 2007–present) and 15 years (Tdap; 2016–present).

The pertussis incidence data from 1996 to 2021 are presented in Fig 1. The data indicate a reduction in the incidence among infants with the rollout of DTaP immunization (0–1 yo; Figs 1A). They also indicate that the initial high incidence of pertussis among 1–10 yo in the early 2000s gave way to increased incidence in individuals older than 10 (Figs 1A and 1B). Since 2015, incidence among those ten years or older accounts for more than 60% of recorded pertussis cases in Sweden (Fig 1B). These data also highlight a notable rise in incidence among the 10+ age group from 2014 onwards. The mean age of confirmed cases has risen from about 6 years in 1997 to 30 years in 2019 (Fig 1C).

The shifting patterns of pertussis incidence need to be understood within the context of immunization in Sweden. As shown in Fig 1D, in 1997 two and three component acellular pertussis vaccines were introduced into the routine immunization schedule at 3, 5 and 12 months. Coverage for the routine schedule is estimated to exceed 97% [52]. In 2007, a booster was introduced for 6 yo (again using a mixture of two and three component acellular vaccines) and in 2016, a booster was introduced for 15 yo using the Pertussis Toxin-only Tdap vaccine. Coverage for these boosters is estimated to be 90% [52]. From 2005 to 2012, Sweden also had a catch-up booster at 10 years of age.

**Fig 2:**
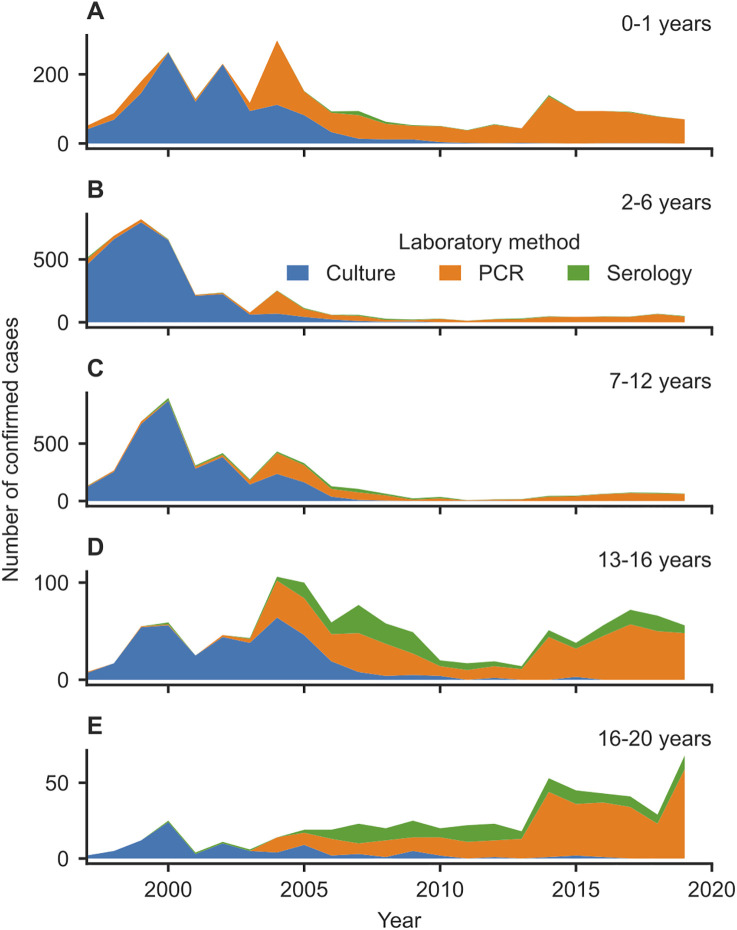
Annual counts of confirmed pertussis cases by testing method. A–E) Number of annual confirmed cases by method used to confirm the case. Data were available for 5 age groupings: [0, 1] years (panel A), [2, 6) years (panel B), [7,12] years (panel C), [13,16] years (panel D) and [16,20] years (panel E). Data for individuals over 20 years were not available.

Pertussis surveillance in Sweden in the early 2000s was primarily based on culture [48,56]. Through time, culture was replaced by serology and especially PCR-based methods. Counts of confirmed cases by age group and testing method are shown in Fig 2. From 2014 onwards, there has been an abrupt spike in the number of cases confirmed via PCR.

**Fig 3:**
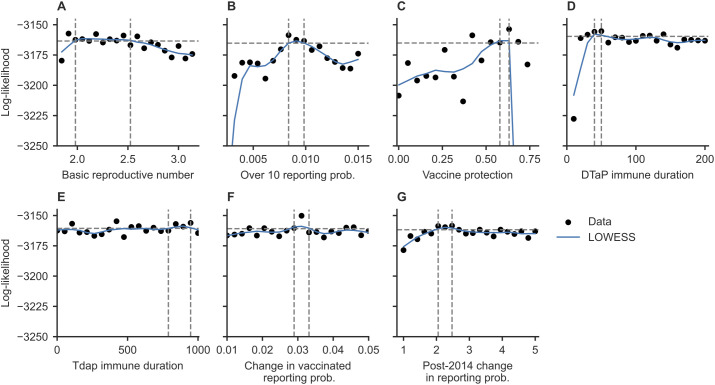
Estimated profile likelihood functions. A–G) Estimated profile likelihood function for the basic reproductive number (panel A), vaccine protection parameter (panel B), over-10 reporting probability (panel C), mean DTaP immune duration (panel D), mean Tdap immune duration (panel E), change in vaccinated reporting probability (panel F) and post-2014 change in reporting probability (panel G). Black points correspond to the estimated profile likelihood found by maximising the all unknown model parameters apart from the profiled parameter, which is fixed to the value shown. Blue lines are calculated using LOWESS smoothing and vertical grey lines indicate the bound of the 95% confidence interval. Our methodology was unable to reliably estimate Tdap immune duration.

Using likelihood-based inference, we fitted our transmission model to the epidemiological dataset (see Methods). Maximum likelihood estimates for unknown model parameters are shown in Table 1. We examined the precision in our estimates by constructing profile likelihood functions for each parameter, with uncertainty quantified using a likelihood-ratio test [57], as shown in Fig 3. We estimated a relatively low value for the basic reproductive number, *R*_0_ = 2.43 with 95% CI (1.98, 2.52) (see Fig 3A). The data were found to be consistent with intermediate values of the vaccine protection parameter, κ, indicating that, prior to immunity waning, vaccinated individuals have a probability κ=0.59 (95% CI (0.58 0.63)) of resisting infection upon exposure. We note that of the parameters shown, κ has the noisiest profile likelihood, so the narrow confidence interval should be interpreted with caution. For the over-10 reporting probability, we estimated $\rho_{4}=0.0091$ (95% CI (0.0084, 0.0098)). This value implies that, among unvaccinated individuals, only about 0.1% of infections are reported. We estimated a mean duration of DTaP immunity of $1/\alpha_{D}=43.6$ years (95% CI (40.0, 50.0)). Our maximum-likelihood estimate (MLE) corresponds to vaccine-derived immunity waning in about 23% of individuals by age 20. The model sharply disfavours mean durations of DTaP immunity shorter than ∼40 years, however we note a more shallow decrease in likelihood as the duration of immunity increases. The data are consistent with a substantial reduction in the reporting probability of infections that breakthrough vaccine-derived immunity, with $p_{w}=0.029$ (95% CI (0.029, 0.033)), translating to about a 30-fold reduction in the probability among vaccinated individuals. Our model estimates that from 2014 onwards the reporting probabilities across age-groups more than doubled (by a factor of *p*_2014_ = 2.19; 95% CI (2.05, 2.47)). Finally, we were unable to reliably estimate the duration of Tdap immunity as a consequence of the parameter’s likelihood profile being largely flat. In other words, we find our model fits were agnostic to values of the duration of Tdap immunity. This finding is unsurprising, as Tdap vaccination only began in 2016, four years before the end of our dataset.

**Table 1:** Maximum-likelihood estimates (MLE) of model parameters. The MLE was found by maximising the likelihood of the age-structured incidence data using our transmission model (see Methods). We estimated 95% confidence intervals (CIs) using the method of Ionides et al. [57]. Note our methodology was unable to reliably estimate the duration of Tdap immunity.

Parameter	Description	MLE and 95% CI
*R*_0_	Basic reproductive number	2.43 (1.98, 2.52)
$1/\alpha_{D}$	Mean DTaP immunity duration	43.6 years (40.0, 50.0)
$1/\alpha_{T}$	Mean Tdap immunity duration	***
κ	Vaccine protection parameter	0.59 (0.58 0.63)
$\rho_{4}$	Over 10 yo reporting probability	0.0091 (0.0084, 0.0098)
$p_{w}$	Change in vaccinated reporting probability	0.029 (0.029, 0.033)
*p*_2014_	Post-2014 change in reporting probability	2.19 (2.05, 2.47)

**Fig 4:**
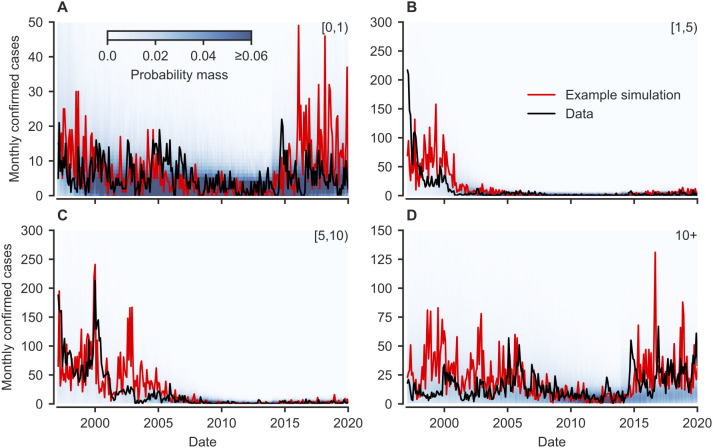
Comparison of simulation output with data. A–D) Probability mass function (PMF) of monthly confirmed cases simulated using the transmission model compared with observed data (black). The PMF was calculated from 1000 independent simulations which were performed using maximum-likelihood estimates of unknown model parameters (see Methods). Also shown is the most similar simulation replicate (red), as ranked using the reporting model (see Methods). Panels correspond to the reporting age groupings: [0, 1] years (panel A), [1, 5) years (panel B), [5, 10) years (panel C) and 10+ years (panel D).

To assess the adequacy of the model at explaining the data, we compared data simulated using the fitted model to the epidemiological surveillance data (Fig 4). Blue shading corresponds to the probability mass estimated from 1000 sampled probabilistic trajectories. The black curve is the observed weekly surveillance data used for parameter inference. Also shown is the simulated trajectory most similar to the observed data (red curve), as calculated via maximising the reporting probability (see Methods). We find that simulated trajectories capture the rapid decline in notifications among [1, 5) and [5, 10) yo individuals (see Fig 4B and 4C). Both the data and simulated trajectories feature decaying oscillations – overall declines in incidence punctuated by resurgences of decreasing magnitude. As expected for a stochastic dynamical system, especially given the long serial interval of pertussis [12,27,58], the timing and magnitude of peaks varies between realisations [27,59,60]. Whether the simulated trajectory captures the recent increase in notifications among 10+ yo individuals (see Fig 4D) depends on whether the stochastic realisation has an upsurge that coincides with the estimated increase in reporting rates post-2014 (see Table 1).

**Fig 5:**
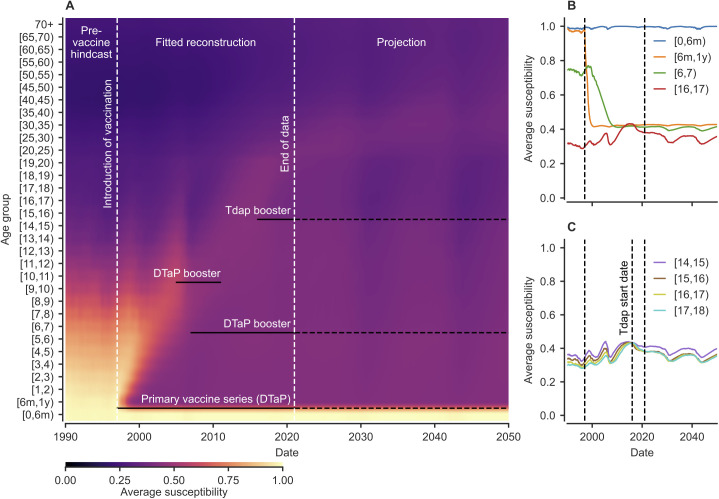
Reconstructed population-level susceptible profile. A) Average susceptibility of each age group in the full model by year. Average susceptibility is defined the proportion of each age group that either i) are immune naive or ii) have incomplete immunity, weighted by the vaccine protection parameter. Simulations are from a single representative replication using the maximum likelihood estimate for uncertain model parameters. Results are shown for three time periods: a pre-vaccination hindcast (before 1997), a fitted reconstruction during the period of data availability (1997–2020) and a projection from 2020 to 2050. For the post-2020 projection, covariates (*e.g.,* birth rate, vaccine schedule and uptake) are kept at their 2020 values. B) A slice of the average susceptibility through time for four age groups. C) A slice of the average susceptibility through time for four teenage age groups. In the model, the Tdap vaccine is administered as individual age from the [14, 15) to [15, 16) age group.

To understand the epidemiological impact of immunization, we used our fitted model to reconstruct the temporal changes in the average susceptibility of each age group. As shown in Fig 5, coincident with the resumption of immunization in 1996 there is a rapid drop in susceptibility among individuals eligible to receive the primary vaccine series; an effect that extends through the population as the vaccinated cohorts age (see Fig 5A and 5B). Due to the high immunization coverage, susceptibility among these individuals is largely due to the leakiness of vaccine protection. Fig 5A also highlights a shallow vertical gradient, which is the net effect of waning immunity and decreases in susceptibility (*i.e.,* increases in immunity) due to transmission. The figure further reveals a small but notable impact of the Tdap booster introduction, by comparing the susceptibility of [14, 15) yo individuals, who are below the scheduled age, with older age groups (Fig 5C). Our results suggest that, despite the absence of vaccine- or maternally-derived immunity, most new born infants remain uninfected (i.e., are not infected) until they are eligible for vaccination (see Fig 5B). This results primarily from the relatively low basic reproduction number that leads to a high mean age at infection.

**Fig 6:**
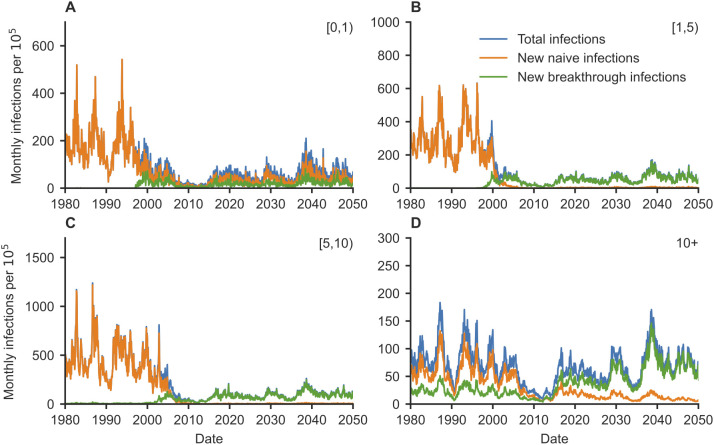
Comparison of breakthrough and immune-naive infections. A–D) Reconstructed total monthly new infections (including both unobserved infections and confirmed cases) by age group per 10^5^. Panels correspond to the reporting age groupings used in the model: [0, 1] years (panel A), [1, 5) years (panel B), [5, 10) years (panel C) and 10+ years (panel D). Note the differences y-axis ranges between panels.

The partially observed Markov process framework [61] adopted here allowed us to reconstruct the monthly infections in the model (both observed and unobserved) broken down by immune status. As demonstrated in Fig 6, following the resumption of infant immunization there was a clear drop in cases across age groups with an abrupt drop in immune naïve infections, as a consequence of high vaccine uptake (Fig 6A–6D). Despite the rise in breakthrough infections across ages, as expected from a leaky vaccine that only provides partial protection against infection, vaccination results in a net reduction in circulation. The resurgence post ∼2005 may be a manifestation of an end-of-honeymoon effect [62]: a legacy of incomplete vaccination with an effective, but imperfect, vaccine against a background of slow demographic turnover. As explained by Riolo *et al.,* [29]: during the first years of immunization, “the population benefits both directly from vaccine protection of children and indirectly from herd immunity established by natural infection in the pre-vaccine era. As cohorts of children born in the vaccine era grow up, the latter effect diminishes and incidence among adults inevitably rises.” An additional dynamical effect of vaccination in Sweden is to disrupt the clear multi-annual cycle observed pre-1997 [21]. Extrapolating from present trends, assuming no changes in vaccination policy, the model predicts a gradual rise in infections. Total infections in 10+ yo individuals are projected to approach pre-vaccine levels by 2050 (Fig 6D), however infections in all other age groups remain substantially reduced (Fig 6A–C).

**Fig 7:**
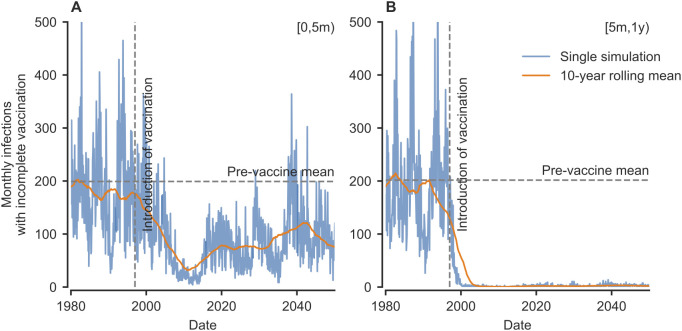
Evaluating indirect protection of unvaccinated newborns. A–B) Reconstructed monthly new infections per 10^5^ in individuals with incomplete vaccine immunity (blue). Also shown is the 5-year rolling mean monthly new infections (orange). Panels correspond to individuals aged [0, 5) months (panel A) and [5, 12) months (panel B). Note that infants in the [0, 5) month age group are too young to have protection from the primary vaccine schedule, in contrast to the [5, 12) month age group, amongst whom vaccine coverage has remained high post-1997 (panel B).

Key to understanding the impact of pertussis transmission is the number of infections in unvaccinated infants [13,22,23,63,64]. Simulating from the fitted model, we found that although infections in unvaccinated individuals rebounded, they remained below the pre-vaccine mean (Fig 7A). For infants over 5m, the high vaccine coverage meant there are very few infections among those unvaccinated (Fig 7B). Our results suggest that indirect protection prevents about half the unvaccinated infections that would otherwise have occurred.

To quantify the overall vaccine impact in Sweden, we used the approach developed by McLean & Blower [65] and extended by Magpantay *et al.* [66,67]. Specifically, the vaccine impact, ω, is defined as


$$\begin{array}{c} \omega =\kappa (1-\phi )(1-\epsilon_{W}), \end{array}$$ (1)

where ϕ, κ, and $\epsilon_{W}$ are respectively the probability of vaccine failure to take (primary failure [44]), vaccine protection against infection [44,65,66], and the probability of immunity waning during a vaccinee’s lifetime (such that $\epsilon_{W}=\alpha_{D}/(\alpha_{D}+\mu )$). The vaccine impact can take values between 0 (the vaccine provides no protection against infection) and 1 (the vaccine provides complete lifelong protection against infection). We estimate vaccine impact to be ω=0.28. As previously demonstrated [66,67], the vaccine impact, ω, modulates the classic immunization eradication threshold [68,69] as follows:


$$\begin{array}{c} v>(1-\frac{1}{R_{0}})(\frac{1}{\omega }), \end{array}$$ (2)

where *v* is the immunization coverage and *R*_0_ is the basic reproduction number. Thus, given our MLE value of *R*_0_ = 2.43 (see Table 1), a vaccine impact of 0.28 confirms the conclusion reached by Domenech de Celles *et al.,* [27] that pertussis cannot be eradicated with existing aP vaccines.

## Discussion

In this paper, we have examined the epidemiological dynamics of pertussis in Sweden since the resumption of routine immunization in 1996. The roll out of the DTaP vaccine coincided with an overall reduction in pertussis incidence across age groups, especially among infants too young to be immunized [21,23]. Since the mid-2010s, however, incidence has increased, especially among those aged 10 and over (Fig 1). To explain these patterns, we confronted incidence reports with a mechanistic, age-stratified, stochastic transmission model using likelihood-based methods. The results of our inference explain these patterns in the data via a combination of a low basic reproductive number (Fig 3A), incomplete (leaky) DTaP-derived immunity (Fig 3B), a much lower reporting probability of infections in older individuals (Fig 3C) and waning of vaccine-derived immunity (Fig 3D). Given our model, this combination of parameters is necessary to explain the initially rapid reduction in pertussis notifications, coupled with the persistence of infections in unvaccinated newborns in the face of high vaccine coverage (see Table 1).

Our estimate of *R*_0_ is considerably lower than values previously estimated. This is in part because prior estimates of the basic reproductive number have either resulted from the use of different methods (including the use of age at first infection estimates [70]; feature matching [10,42,60] and trajectory matching [28]) or data from different populations (*e.g.,* USA [26,27,70], the UK [42], Italy [67] or Australia [10]). Of the previous studies, perhaps the most comparable in approach is a study of pertussis in Massachusetts [27]. We recognise a number of epidemiological differences between the Massachusetts study and ours. Crucially, unlike in Massachusetts, the roll-out of vaccination in Sweden was abrupt, as it was a new vaccine component introduced to a pre-existing immunization program with high uptake (estimated to exceed 97%). Additionally, transient effects may have played a role in Sweden, given the cessation of a whole cell pertussis vaccination campaign 17 years prior to 1997. Furthermore, unlike in Massachusetts, our data did not encompass the pre-vaccine era, meaning our best-fitting parameters may only be appropriate after the roll out of the DTaP vaccine. We submit that a fuller understanding of these issues would require studies that additionally account for serological survey data [71], to estimate the prevalence of pertussis immunity prior to the introduction of vaccination in 1997. We caution however that there are challenges in interpreting pertussis serological data [72].

While our work supports the conclusion that the vaccine impact is insufficient for the population to achieve herd immunity [27], our fitted model provides evidence for indirect protection of unvaccinated newborns. We estimate that, due to declines in the overall prevalence of pertussis in Sweden, there is about a 50% reduction in infections among this most at-risk age group. This reduction is consistent with a study of pertussis incidence in Washington state, USA [43]. Given our estimate of the vaccine protection parameter and the already high vaccine uptake, we suspect that the addition of further booster doses is unlikely to increase the indirect protection afforded to infants.

Our fitted model explains the apparent resurgence of pertussis in Sweden as a confluence of two factors. The first, known as the honeymoon effect [29,62], is a dynamical transient that follows a rapid decrease in the average population susceptibility (*e.g.,* due to a sudden increase in vaccination). A reduction in prevalence (the honeymoon) gives way to a rebound and stabilisation at the lower vaccine-mediated endemic equilibrium. The second, is an increase in the infection reporting probability from 2014 onwards, which we estimate to have approximately doubled (*p*_2014_ = 2.19). We suspect the second explanation has its roots in the shift in case ascertainment methodologies, from primarily using bacterial culture to the more sensitive PCR (see Fig 2). In particular, it is important to note that in individuals older than 13 years of age, a marked increase in confirmed pertussis using PCR was observed starting in 2014 but not with serology or culture (Fig 2E).

Our results reflect the perennial challenge in interpreting pertussis epidemiological data: disentangling the confounding effects of changes in transmission rates from changes in disease reporting [9,73]. Our modelling made use of a sophisticated, age-specific and immune-status specific reporting model in an attempt to achieve this. We aimed to account for changes in the case confirmation process through time (via an increase in the infection reporting probability post-2014, which was inferred from data). Data on the number of cases confirmed by test method (Fig 2), show a clear shift from the use of bacterial culture to PCR from 2004 onwards. PCR tests are recognised as a more sensitive diagnostic method [74], consistent with our observed increase in case reports. The roughly threefold increase in cases detected by PCR in 2014, but not by serology or culture, may reflect a change in diagnostic procedures (such as cycle thresholds or changes in primers). Since we have been unable to obtain data on the number of tests performed by age group (the denominator in diagnostic method percent positive calculations), we cannot establish whether the observed increase in reported cases by PCR starting in 2014 (Fig 2) is due to increased transmission or changes in laboratory diagnostics.

Our results suggest that infections of vaccinated individuals are much less likely to be confirmed by laboratory testing. During the 2010s, the data are predominantly picking up two population subgroups: newborn infants who are too young to have received vaccine-derived immunity, and older (over 10y) immune naive individuals who were too old to be vaccinated but had not previously been infected (in part due to declining circulation). Information of the vaccine status of confirmed cases would enable us to verify these findings.

Uncertainty was quantified using the profile likelihood approach of Ionides et al. [57]. As mentioned previously, we were unable to estimate the duration of Tdap immunity due to the flat profile likelihood, which we attributed to the short period of data available after the introduction of the booster dose in 2016. Of the model parameters, the vaccine protection had the noisiest profile likelihood. While the upper bound on the confidence interval appears well identified the lower bound is less clear. However, this issue does not affect our main finding, namely that vaccine impact is insufficient for herd immunity, as lower values of vaccine protection only reduce the vaccine impact further. Another limitation was our inability to quantify the correlation between estimates of model parameters due to the prohibitive computational costs required. Calculating this correlation structure is necessary for future efforts to quantify uncertainty in the estimated vaccine impact; inspecting scatter plots of parameter estimates obtained from estimating the profile likelihood function suggests three parameters are likely correlated: the basic reproductive number, over-10 reporting probability and vaccine protection (see S4 Fig). That said, our confidence intervals remain reliable quantifications of individual parametric uncertainty as the are constructed using Wilks’ theorem, which is valid for correlated model parameters (see [75]).

One subtle but important point in estimating the duration of immunity is whether the estimate was conditioned on survival or not. Studies of vaccine immune duration are based on recording the time at which a participant loses immunity [76]. Therefore, estimates of the duration of immunity from such survey data are conditioned on participant survival. In contrast, our statistical inference estimates the unconditional duration of immunity, i.e., independent of whether an individual survives long enough to lose protection. To reconcile the two different estimated quantities, we also estimated the conditional mean duration of immunity, finding it to be 34.0 years (95% CI (32.6, 36.0)) (see Methods for details).

The COVID-19 pandemic resulted in substantial global disruption to human society and also co-circulating infectious diseases [77]. Our dataset, and therefore statistical inference, ends at the start of 2020 meaning we were unable to study the pandemic’s effects. Sweden presents a compelling case study, as the country adopted less stringent control measures compared with other neighbouring countries, for instance Denmark, Finland and Norway [78]. It would be interesting to investigate whether these differences in pandemic response are manifested in different pertussis dynamics. Furthermore, since 2023, we have witnessed a dramatic resurgence in pertussis notifications in a number of countries, including Sweden. The extent to which this can be attributed to loss-of-immunity during the pandemic is a pressing research question. Disruption due to the pandemic is expected to be transitory (see [77]), and therefore not have a substantial effect our long-term projections (Figs 5–7).

Unfortunately, pertussis continues to circulate in a number of countries, including Sweden, despite high vaccine coverage. Our results suggest that immunization is successfully affording indirect protection to newborn infants who are too young to be vaccinated. That said, consistent with prior studies [27], our statistical inference demonstrates that pertussis elimination is not possible with routine immunization using acellular vaccines due to the combined effects of vaccine leakiness and waning immunity. A fruitful research avenue, therefore, is the use of transmission models that have been rigourously fit to data in identifying appropriate age-specific booster strategies [79,80].

## Methods

### Data

#### Incidence data.

Monthly age-stratified incidence data were sourced from the Public Health Agency of Sweden. The data cover the time interval from 1997-01-01 to 2020-12-31.

#### Contact matrix and demographic data.

We used the *socialmixr* R package to construct the contact matrix. We used the POLYMOD survey data from the United Kingdom [54], adjusted with demographic data from Sweden. The contact matrix was constructed using 2-year age ranges from age 0 years to 20 years, 5-year ranges from 20 years to 70 years, and all others in the 70 + age class. Following Domenech de Cellès *et al.* [27], we used an augmented contact matrix in the model itself, where infants were split into two age groups, [0, 5) months and [5, 12) months, and age groups were in 1-year ranges from 1 to 20 years.

The annual birth rate in Sweden was parameterised using demographic data from Official Statistics Sweden [81,82].

### Model

To model the spread of pertussis in Sweden, we formulated an age-structured transmission model. The model has the structure of the classic Susceptible-Exposed-Infectious-Recovered-Vaccinated (*SEIRV*) model [69], with modifications to account for i) different mechanisms of immune failure and ii) gamma-distributed durations of vaccine-derived immunity [42]. To account for demographic stochasticity in the disease transmission process, we formulated our model as a discrete time-Markov Chain (DTMC), along the lines of the chain-binomial model tracking the probabilities of each individual in the system transitioning between compartments [59,69]. Individuals in our model are subdivided by age into 32 age groups, [0, 5) months, [5, 12) months, then 1 year ranges from 1 to 20 years, then 5 year ranges until 70 years, with a final group for 70 + years. The rate at which individuals transition from age group *i* to *i* + 1, $\mu_{i}$, is given by the inverse of the age group range. Individuals age out of the final group with rate $\mu_{32}=1/5\;{\text{years}}^{-1}$, giving a mean life expectancy of 75 years. The birth rate, ν(t), is parameterised using demographic data (see previous section). Vaccines are modelled as being administered as individuals reach the ages given in the vaccine schedule, and therefore coupled with the ageing process. We modelled the duration of vaccine derived immunity as following a 2 stage Erlang distribution, with the mean duration of DTaP and Tdap immunity given by $1/\alpha_{D}$ and $1/\alpha_{T}$ respectively. We chose this disruption as it is peaked around the mean duration. Given the importance of immune failure to our study, we explicitly track whether infections occur in individuals who are unvaccinated (compartments with a superscript *u*) or have some previous immunity (superscript *w*). Infection follows the standard *SEIR*-scheme: upon exposure to the pathogen, individuals move into the exposed compartment, then after a latent period with mean 1/σ they move into the infectious compartment. After the infectious period, with mean 1/γ, infectious individuals transition into the recovered compartment.

In total our model has 11 different compartments. For immune naïve individuals there are susceptible ($S^{u}$), exposed ($E^{u}$) and infectious ($I^{u}$) compartments before they move to the recovered (*R*) compartment. For individuals who have successfully received some degree of protection from vaccination we have four model compartments, corresponding to each of the two vaccines and the two stages of the Erlang distribution: $V^{D,1}$, $V^{D,2}$, $V^{T,1}$ and $V^{T,2}$ where *D* and *T* denote DTaP and Tdap respectively. For individuals whose immunity has waned we have a susceptible compartment $S^{w}$, and then exposed and infectious compartments for breakthrough infections, $E^{w}$ and $I^{w}$ respectively.

The deterministic skeleton of the model [27,69], found in the continuous time limit Δt→0 is given by a system of coupled ODEs,


$$\begin{array}{c} \frac{dS_{i}^{u}}{dt}=\nu_{i}(t)-\lambda_{i}(t)S_{i}^{u}-\mu_{i}S_{i}^{u}+(1-\chi_{i-1})\mu_{i-1}S_{i-1}^{u}, \end{array}$$ (3)

$$\begin{array}{c} \frac{dE_{i}^{u}}{dt}=\lambda_{i}(t)S_{i}^{u}-\sigma E_{i}^{u}-\mu_{i}E_{i}^{u}+\mu_{i-1}E_{i-1}^{u}, \end{array}$$ (4)

$$\begin{array}{c} \frac{dI_{i}^{u}}{dt}=\sigma E_{i}^{u}-\gamma I_{i}^{u}-\mu_{i}I_{i}^{u}+\mu_{i-1}I_{i-1}^{u}, \end{array}$$ (5)

$$\begin{array}{c} \frac{dR_{i}}{dt}=\gamma \left(I_{i}^{u}+I_{i}^{w}\right)-\alpha_{R}R_{i}-\mu_{i}R_{i}+\mu_{i-1}R_{i-1}, \end{array}$$ (6)

$$\begin{array}{cc} \frac{dV_{i}^{D,1}}{dt} & =-(1-\kappa )\lambda_{i}(t)V_{i}^{D,1}-2\alpha_{D}V_{i}^{D,1}-\mu_{i}V_{i}^{D,1}\\ & \;+\chi_{i-1}(t)\mu_{i-1}\left(S_{i-1}^{w}+V_{i-1}^{D,2}\right)+\mu_{i-1}V_{i-1}^{D,1} \end{array}$$ (7)

$$\begin{array}{cc} \frac{dV_{i}^{D,2}}{dt} & =2\alpha_{D}V_{i}^{D,1}-(1-\kappa )\lambda_{i}(t)V_{i}^{D,2}-2\alpha_{D}V_{i}^{D,2}-\mu_{i}V_{i}^{D,2}\\ & \;+\left(1-\chi_{i-1}(t)-\psi_{i-1}(t)\right)\mu_{i-1}V_{i-1}^{D,2} \end{array}$$ (8)

$$\begin{array}{cc} \frac{dV_{i}^{T,1}}{dt} & =-(1-\kappa )\lambda_{i}(t)V_{i}^{T,1}-2\alpha_{T}V_{i}^{T,1}-\mu_{i}V_{i}^{T,1}\\ & \;+\psi_{i-1}(t)\mu_{i-1}\left(S_{i-1}^{w}+V_{i-1}^{D,2}\right)+\mu_{i-1}V_{i-1}^{T,1} \end{array}$$ (9)

$$\begin{array}{c} \frac{dV_{i}^{T,2}}{dt}=2\alpha_{T}V_{i}^{T,1}-(1-\kappa )\lambda_{i}(t)V_{i}^{T,2}-2\alpha_{T}V_{i}^{T,2}-\mu_{i}V_{i}^{T,2}+\mu_{i-1}V_{i-1}^{T,2} \end{array}$$ (10)

$$\begin{array}{cc} \frac{dS_{i}^{w}}{dt} & =\alpha_{R}R_{i}+2\alpha_{D}V_{i}^{D,2}+2\alpha_{T}V_{i}^{T,2}-\lambda_{i}(t)S_{i}^{w}-\mu_{i}S_{i}^{w}\\ & \;+\left(1-\chi_{i-1}(t)-\psi_{i-1}(t)\right)\mu_{i-1}S_{i-1}^{w}, \end{array}$$ (11)

$$\begin{array}{cc} \frac{dE_{i}^{w}}{dt} & =\lambda_{i}(t)S_{i}^{w}+(1-\kappa )\lambda_{i}(t)\left(V_{i}^{D,1}+V_{i}^{D,2}+V_{i}^{T,1}+V_{i}^{T,2}\right)\\ & \;-\sigma E_{i}^{w}-\mu_{i}E_{i}^{w}+\mu_{i-1}E_{i-1}^{w}, \end{array}$$ (12)

$$\begin{array}{c} \frac{dI_{i}^{w}}{dt}=\sigma E_{i}^{w}-\gamma I_{i}^{w}-\mu_{i}I_{i}^{w}+\mu_{i-1}I_{i-1}^{w}, \end{array}$$ (13)

for i=1,2,…,32. The birth rate $\nu_{i}(t)=\delta_{i,1}\nu (t)$ where we use the Kronecker delta, $\delta_{i,j}=1$ if *i* = *j* and 0 otherwise. The functions $\chi_{i}(t)$ and $\psi_{i}(t)$ denote the probability that an individual ageing from *i* to *i* + 1 at time *t* recieves a dose of the DTaP and Tdap vaccine respectively (see next section for details). See S1 Fig for a model diagram.

The force of infection, $\lambda_{i}(t)$, is given by


$$\begin{array}{c} \lambda_{i}=q_{i}\sum_{j=1}^{32}F_{ij}(t)C_{ij}\frac{I_{j}^{u}+I_{j}^{w}+\iota }{N_{j}} \end{array}$$ (14)

where $q_{i}$ is the susceptibility and ι the rate cases are imported to the population, $N_{j}$ is the total population size of age group *j* (found by summing over compartments) and the contact matrix element $C_{i,j}$ gives the rate at which an individual in age class *i* contact individuals in age class *j*. The matrix with elements $F_{ij}(t)$ gives the seasonal forcing to transmission (with period 1 year) which is modelled using a set of three basis splines, see [27,83]. Simulations were initialised in a fully susceptible population at *t* = 1900 to allow for a burn-in period before the start of the incidence data in 1997.

To relate our model output to the epidemiological data, which are periodically released counts of total new confirmed cases by age, we included an observation process. In order to study the effects of vaccination, we separately recorded the number of new immune naive and breakthrough infections in time interval [t−Δ,t), denoted $C_{i,t}^{u}$ and $C_{i,t}^{w}$ respectively. Dynamically, these quantities were defined as the total number of new infection events (i.e., transitions into $E_{i}^{u}$ and $E_{i}^{w}$) during the time interval. To reduce the dimensionality of the observed data (and therefore make particle filtering computationally feasible), we further aggregated case counts into four more coarsely resolved age groups: (0, 1) years, (1, 5) years, [5, 10) and 10+ , informed by a previous study [27]. The four age groups were each assigned a separate case reporting probability, $\rho_{i}$ due to widely documented age-dependence in pertussis symptomatology and case ascertainment [27]. We modelled the probability for the number of observed cases, $Y_{i,t}$, as following a negative binomial distribution with probability density function $f(y_{i,t};\mu_{i,t},1/\tau )$ where τ is the dispersion parameter and $\mu_{i,t}$ is the expected number of observed cases, given by


$$\begin{array}{c} \mu_{i,t}=\pi_{2014}(t)\rho_{i}(C_{i,t}^{u}+p_{w}C_{i,t}^{w}). \end{array}$$ (15)

Here, $p_{w}$ is the ratio of the reporting probability of an infection in a vaccinated individual relative to in an immune naive individual. The factor $\pi_{2014}(t)$ accounts for possible changes in the reporting probability post-2014 (see Figs 1 and 2), and is equal to 1 pre-2014 and *p*_2014_ afterwards. Note that in the interests of model parsimony we assume than any changes in reporting probability post-2014 apply to all age-group equally. The variance in observed cases is given by $\sigma_{i,t}^{2}=\mu_{i,t}+\tau \mu_{i,t}^{2}$.

Simulation projections were performed by simulating our model forwards in time using MLEs for unknown model parameter values (see next section). Covariates were fixed to their final (end of 2019) values. We excluded the effects of the COVID-19 pandemic from our projections because a) our incidence dataset ended in 2020 meaning we were unable to precisely quantify transient pandemic-associated effects and b) we were interested in generating long-term projections (i.e., after the effects of the pandemic have dissipated, for details see [77]).

#### Vaccination coverage and schedule.

Within the model, whether individuals receive a vaccination or not was a product of two terms, first the vaccine uptake probability for a given dose and second whether the dose was in the vaccine schedule at the time the individual was eligible for vaccination. Both terms were parameterised using vaccine coverage data. Taking into account changes in the vaccine schedule, our model included four different scheduled vaccination events (considering the three dose primary vaccine series as a single event administered at age 5 months). Individuals were modelled as receiving vaccination as they age out of the age group indicated in the vaccine schedule. Throughout the time period of study there were four different age groups that received vaccines. The 3-dose DTaP course was available to infants (*i* = 1) from 1995 to 2020, i.e., the entire time span of the data. Five year olds, *i* = 5, received a DTaP booster from 2007 to 2020. Ten year olds, *i* = 10, received a DTaP booster from 2005 to 2011. A Tdap booster was made available for 15–16 year olds, *i* = 15, from 2016 to 2020. Due to the availability of time series data, the primary vaccine series vaccination probability for infants was included as a year-specific covariate, *v*_1_(*t*). Due to limited available data and noting only small interannual variation, we used a fixed vaccination probability for the three booster doses, $v_{i}$ for *i* = 5, 10 and 15 during the period each dose was administered and 0 otherwise (see Table 2). The remaining age-groups did not receive vaccination, i.e., $v_{i}=0$ for all time. Note that the timing of inclusion in the vaccine schedule mean that most individuals will not have been eligible for all four vaccination events. The probability of primary vaccine failure (i.e., it has no effect on the recipient) is denoted by $\phi_{D}$ and $\phi_{T}$ for DTaP and Tdap respectively.

**Table 2:** Model parameters and values. For estimated parameters, the interval shown is the range of initial guesses used in the Nelder-Mead optimisation. The importation rate was set to a small but non-zero value to prevent stochastic extinction (see [21,27]). The seasonality basis spline coefficients were set to match pertussis seasonality patterns (see [27]). The mean duration of natural immunity was set based on the findings of previous studies [42,86].

Parameter	Description	Start range
*q*_1_	Susceptibility factor	[0, 0.1]
ι	Importation rate	0.01 days^-1^
1/σ	Mean latent period duration	8 days [27]
1/γ	Mean infectious period duration	15 days [27]
$1/\alpha_{R}$	Mean natural immunity duration	130 years
$1/\alpha_{D}$	Mean DTaP immunity duration	[10, 100] years
$1/\alpha_{T}$	Mean Tdap immunity duration	[0, 1000] years
$\phi_{D}$	Probability of primary vaccine failure, DTaP	0.01 [84]
$\phi_{T}$	Probability of primary vaccine failure, Tdap	0.2 [84]
κ	Vaccine protection parameter	[0, 1]
*b*_1_	Basis spline coefficient	-0.58
*b*_2_	Basis spline coefficient	-0.62
*b*_3_	Basis spline coefficient	-0.16
$\rho_{1}$	Reporting probability, under 1 years	0.15 [27]
$\rho_{2}$	Reporting probability, [1, 5) years	0.09 [27]
$\rho_{3}$	Reporting probability, [5, 10) years	0.04 [27]
$\rho_{4}$	Reporting probability, 10+ years	[0, 0.02]
τ	Observation process dispersion parameter	0.27 [85]
$p_{w}$	Ratio of vaccinated to unvaccinated reporting probabilities	[0, 2]
*p*_2014_	Relative change in reporting probability post-2014	[1, 5]
*v*_1_(*t*)	Primary vaccine series coverage	Covariate data [52]
*v*_2_	Pre-school DTaP booster coverage	0.92 [52]
*v*_3_	10 yo DTaP booster coverage	0.91 [52]
*v*_4_	15 yo Tdap booster coverage	0.91 [52]
ν(t)	Birth rate	Covariate data [81,82]

Together, the probability that an individual who ages from age group *i* to age group *i* + 1 at time *t* is successfully vaccinated with DTaP is given by


$$\begin{array}{c} \chi_{i}(t)=\sum_{j\in \{1,5,10\}}\delta_{i,j}\phi_{D}v_{j}(t){𝕀}_{T_{j}}, \end{array}$$ (16)

where $\delta_{i,j}$ is the Kronecker delta, ${𝕀}_{T_{j}}$ is the indicator function and $T_{j}$ is the period of time vaccination at age *j* was included in the schedule. Similarly, for Tdap,


$$\begin{array}{c} \psi_{i}(t)=\delta_{i,15}\phi_{T}v_{15}(t){𝕀}_{T_{15}}. \end{array}$$ (17)

### Statistical inference

To estimate unknown model parameters, we fit our transmission model to epidemiological data using likelihood-based inference. The likelihood function for the observed data $\{{𝐲}_{t}{\}}_{t=1}^{T}$ was given by


$$\begin{array}{c} \ell (\theta )=𝔼\left[\prod_{t=1}^{T}\prod_{i=1}^{4}f(y_{i,t};\mu_{i,t},\tau )\right], \end{array}$$ (18)

where the expectation is calculated over the space of model sample paths. As our transmission model was a Markov process which does not possess an analytical solution, we used particle filtering to calculate a numerical Monte Carlo approximation to the likelihood function [87]. Particle filtering provides a computationally efficient way of numerically approximating Eq. 18 by using the reporting probability at each time step, $\prod_{i=1}^{4}f(y_{i,t};\mu_{i,t},\tau )$, to perform a weighted resampling of the set of simulated trajectories, ensuring that trajectories provide dense samples from the region of state space around the data, rather than the full space. Note that we did not use a related method, iterated filtering, to explore the parameter space and maximise the likelihood [61].

Instead, we maximised the likelihood function over the space of unknown parameters using the Nelder-Mead algorithm. To ensure convergence to the global maximum, we performed the optimisation in two steps. First, we performed 150 independent searches for 200 Nelder-Mead iterations, with the likelihood function calculated using 1000 particles in the particle filter. Initial conditions were uniformly sampled from ranges given in Table 2. Next, we used the maxima found from the first round as initial conditions for a second set of 7 independent searches, for 500 Nelder-Mead iterations using 10000 particles. See S2 and S3 Figs. for inference trace plots.

To quantify uncertainty in our parameter estimates, we estimated the profile likelihood function, $\ell_{p}(x)$, for each unknown model parameter $\theta_{p}$. The profile likelihood function is defined as the maximum of the likelihood function over the space of unknown parameters subject to the profiled parameter being fixed to the specified value $\theta_{p}=x$, i.e., $\ell_{p}(x)=\max_{\theta }\ell (\theta ){|}_{\theta_{p}=x}$. For each profiled parameter, we numerically estimated $\ell_{p}(x)$ for a set of 20 evenly spaced values, from which we then computed 95% confidence intervals using a likelihood-ratio test [75], following the method of Ionides et al. [57]. Estimation entailed repeating the maximisation using 20000 particles and 500 Nelder-Mead iteration with the profiled parameter fixed to $\theta_{p}=x$ throughout the optimisation.

### Reconstructing unobserved states

Using our model parametrised using the maximum-likelihood estimates, we sought to reconstruct the 11 unobserved process model compartments, $X_{i,t}=\{S_{i,t}^{u},E_{i,t}^{u},\ldots ,I_{i,t}^{w}\}$. To achieve this, we used Bayes’ theorem to calculate the probability of observing a sample path of the unobserved states, $Q=\{X_{i,t}{\}}_{t=1;\;i=1}^{T;\;4}$, given the observed data, $\{{𝐲}_{t}{\}}_{t=1}^{T}$,


$$\begin{array}{c} P(Q|\{{𝐲}_{t}{\}}_{t=1}^{T})=\frac{\prod_{t=1}^{T}\prod_{i=1}^{4}f(y_{i,t};\mu_{i,t},\tau )P(Q)}{𝔼\left[\prod_{t=1}^{T}\prod_{i=1}^{4}f(y_{i,t};\mu_{i,t},\tau )\right]}, \end{array}$$ (19)

where $\mu_{i,t}$ is determined by the value of the sample path, see Eq. 15.

As a point estimate for the unobserved states, we used the most similar sample path, defined as the sample path for which the probability $P(Q|\{{𝐲}_{t}{\}}_{t=1}^{T})$ is maximised. By generating *M* sample paths from the process model, indexed by m=1,…,M, the most similar sample path, indexed $\hat{m}$, was found numerically,


$$\begin{array}{cc} \hat{m} & =\underset{m\in \;1,\ldots ,M}{\text{arg max}}P(Q_{m}|\{{𝐲}_{t}{\}}_{t=1}^{T})\\ & =\underset{m\in \;1,\ldots ,M}{\text{arg max}}\prod_{t=1}^{T}\prod_{i=1}^{4}f\left(y_{i,t};\mu_{i,t}^{\left(m\right)},\tau \right), \end{array}$$ (20)

where $\mu_{i,t}^{\left(m\right)}$ is the value of $\mu_{i,t}$ for sample path *m*. Note that in Eq. 20 we dropped the constant denominator as it does not alter the location of the maximum.

### Calculating the conditional mean immune durations

Our model parameters $1/\alpha_{R}$, $1/\alpha_{D}$ and $1/\alpha_{T}$ correspond to the mean of the immune duration distributions for natural, DTaP-derived and Tdap-derived immunity respectively. These model distributions are not conditioned on survival of an individual and therefore can have means that exceed human lifespans. To compare our fitted estimate directly with epidemiological surveys of immune duration requires conditioning on participant survival, an obvious precondition for study participation (see [85] for details). In our modelling, we used an approximately constant lifespan, τ=75 years and used a gamma distribution to model the durations of immunity. After some algebra (along the lines of [85]), the conditional mean duration of DTaP immunity can be shown to follow the relationship


$$\begin{array}{c} 𝔼[t|t<\tau ]=\frac{F(\tau ;L+1,L\alpha_{D})}{F(\tau ;L,L\alpha_{D})}𝔼[t] \end{array}$$ (21)

where $𝔼[t]=1/\alpha_{D}$ is the unconditional mean and F(τ;L,Lα) is the cumulative density function of the gamma distribution with shape parameter *L* and rate parameter Lα. Analagous results can be derived to Tdap and natural immunity.

## Supporting information

S1 FigModel diagram.A) Our model has 11 different compartments which are sub-divided into 32 age compartments, indexed i=1,…,32. Individuals move between model compartments via a set of transitions (e.g., infection, recovery and immune waning) as indicated by arrows. The corresponding transition rate is shown next to each arrow. B-C) Individuals age from age group *i* to *i* + 1 with rate $\mu_{i}$. Vaccination is administered as individuals age into an age-group corresponding the the vaccination schedule (see Methods). The probability an individual is successfully vaccinated is $\chi_{i}(t)$. Panels B and C show possible transitions due to DTaP and Tdap vaccination respectively. For all other compartments individuals are unaffected by vaccination, and therefore age from *i* to *i* + 1 with no probability of transitioning to other model compartments.(TIFF)

S2 Fig Trace plot for first round of optimization. A) Estimated log-likelihood function at each iteration of the Nelder-Mead optimization algorithm (see Eq. 19). Lines correspond to each of the 150 independent searches (see Methods). B-H) Trace plots for each of the estimated model parameters.
(TIFF)

S3 Fig Trace plot for second round of optimization. Panels are the same as those shown for the first round (see S2 Fig). Each search is initialised using the best performing runs from round one (see methods). At the end of round two the searches converges onto a smaller region of parameter space with higher log-likelihood.
(TIFF)

S4 FigProfiled parameter scatter plots.Panels show the values of non-profiled model parameters corresponding to the profile likelihood functions (see Fig 3). Specifically, $\theta_{i,p}(c)$ is the value of the parameter $\theta_{i}$ that maximises the likelihood function when parameter $\theta_{p}$ is fixed to the value $\theta_{p}=c$ (see Methods). Note that for all parameter combinations considered, there is a unique maximum with no degeneracy. For the purple (orange) points, the log-likelihood is maximised with the x-axis (y-axis) parameter fixed to the value indicated. The y-axis (x-axis) parameter value is estimated via the likelihood maximisation. Colour shade indicates the value of the profile likelihood function relative to the maximum. The two sets of points are both subsets of the same two-dimensional likelihood surface, and indicate the locations of ridges. A complete grid search would fill in the gaps.(TIFF)
